# Association of maternal body mass index with hemodynamic and vascular alterations at 35–37 weeks' gestation

**DOI:** 10.1002/uog.29170

**Published:** 2025-01-15

**Authors:** M. Charakida, C. Chatzakis, L. A. Magee, A. Syngelaki, T. Mansukhani, P. von Dadelszen, K. H. Nicolaides

**Affiliations:** ^1^ Harris Birthright Research Centre for Fetal Medicine King's College Hospital London UK; ^2^ School of Biomedical Engineering and Imaging Sciences King's College London London UK; ^3^ Institute of Women and Children's Health, School of Life Course and Population Sciences King's College London London UK

**Keywords:** augmentation index, cardiac output, central blood pressure, hemodynamic, obesity, pulse‐wave velocity, total peripheral resistance, uterine artery Doppler

## Abstract

**Objective:**

Globally, one in four pregnant women is classified as overweight or obese, based on their prepregnancy body mass index (BMI). Obese pregnant women are at increased risk of adverse pregnancy outcomes and long‐term cardiovascular disease that occurs earlier in life. This study aimed to assess maternal hemodynamic and vascular parameters at 35–37 weeks' gestation, to understand the alterations that may occur in association with increased maternal BMI and gestational weight gain, and to evaluate obesity‐related pregnancy outcomes.

**Methods:**

This was a prospective observational study of 11 731 women with a singleton pregnancy attending for a routine hospital visit at 35 + 0 to 36 + 6 weeks' gestation at King's College Hospital, London, UK, between December 2021 and June 2024. Women were categorized based on their BMI at 11–13 weeks' gestation, as normal weight (BMI, 18.5–24.9 kg/m^2^), overweight (BMI, 25.0–29.9 kg/m^2^) or obese (BMI, ≥ 30 kg/m^2^). We recorded details regarding maternal demographic characteristics and medical history, used Doppler ultrasound to assess the uterine artery pulsatility index (UtA‐PI) (as a marker for uteroplacental perfusion) and ophthalmic artery peak systolic velocity (PSV) ratio (as a marker for small vessel peripheral circulation), and measured carotid‐to‐femoral pulse‐wave velocity, augmentation index (as direct and indirect markers of aortic stiffness, respectively), cardiac output, total peripheral resistance (TPR), and central systolic and diastolic blood pressure. Multivariable analysis was performed to examine the relationship of BMI and gestational weight gain with hemodynamic and vascular measures, adjusting for maternal demographics, medical history, pregnancy characteristics and pregnancy outcomes (including pre‐eclampsia and gestational diabetes mellitus).

**Results:**

Overweight and obese women were more often of black ethnicity, and had higher central systolic and diastolic blood pressure, cardiac output, aortic stiffness and UtA‐PI, compared with normal‐weight women. There was no significant difference between overweight or obese women and normal‐weight women with regard to TPR and ophthalmic artery PSV ratio. On multivariable analysis, increasing BMI at 11–13 weeks and gestational weight gain between 11–13 weeks and 35–37 weeks were independently associated with increases in all cardiovascular indices (including ophthalmic artery PSV ratio), apart from TPR.

**Conclusions:**

Women with a high BMI in early pregnancy *vs* normal‐weight women, and those with higher gestational weight gain, had worse maternal hemodynamic and vascular indices at 35–37 weeks' gestation, independent of baseline and pregnancy characteristics. Our findings support the notion that optimization of prepregnancy weight and gestational weight gain may improve maternal hemodynamics and vascular function during pregnancy, and therefore may improve pregnancy outcomes. © 2025 The Author(s). *Ultrasound in Obstetrics & Gynecology* published by John Wiley & Sons Ltd on behalf of International Society of Ultrasound in Obstetrics and Gynecology.

## INTRODUCTION

In recent years, the global prevalence of overweight and obesity has continued to rise, both among adults overall and specifically in women of reproductive age^1^, ^2^. At present, approximately one in four pregnant women are classified as overweight or obese, based on their prepregnancy body mass index (BMI)^3^. These statistics are of great concern given the association of obesity with an increased risk of adverse pregnancy outcomes and long‐term cardiovascular morbidity, particularly in early life^4^, ^5^.

It is unclear what mediates the relationship between high maternal BMI and adverse pregnancy outcomes. Understanding the hemodynamic adaptations that occur in pregnancies complicated by overweight or obesity may help inform optimal management. A small cross‐sectional study found that pregnant women at term with BMI ≥ 35 kg/m^2^, *vs* those with BMI < 30 kg/m^2^, had higher cardiac output, lower total peripheral resistance (TPR), altered cardiac geometry and a degree of diastolic and systolic dysfunction^6^. Similarly, a longitudinal study of 232 obese women documented hemodynamic changes with increasing gestational age; obese women had high cardiac output and low TPR in the first trimester, but by the third trimester, cardiac output had decreased and TPR had normalized^7^. These data are gathered from small populations of women, lacking diversity in ethnicity and in other demographic characteristics, making the generalizability of these findings questionable.

The aim of this study was to assess the hemodynamic and vascular indices in a large screening population of women with a singleton pregnancy at 35–37 weeks' gestation, to understand the alterations that may occur in association with increased maternal BMI at 11–13 weeks' gestation and maternal weight gain between 11–13 and 35–37 weeks, and to evaluate obesity‐related pregnancy outcomes.

## METHODS

### Study design and participants

This was a prospective observational study of women attending for a routine hospital visit at 35 + 0 to 36 + 6 weeks' gestation at King's College Hospital, London, UK, between December 2021 and June 2024. The visit included: first, recording of maternal demographics and medical history; second, ultrasound examination for fetal anatomy and growth; third, Doppler imaging of the uterine and ophthalmic arteries; and fourth, measurement of the carotid‐to‐femoral pulse‐wave velocity (PWV). Gestational age was determined by measurement of the fetal crown–rump length at 11–13 weeks or fetal head circumference at 19–24 weeks^8^. Women gave written informed consent to participate in the Advanced Cardiovascular Assessment in Pregnancy study (reference: 18/NI/0013; Integrated Research Application System ID: 237936) which was approved by the NHS research ethics committee.

The inclusion criteria for this study were singleton pregnancy delivering a non‐malformed liveborn neonate. Pregnancies with maternal BMI < 18.5 kg/m^2^, fetal aneuploidy or major fetal abnormality were excluded. Women were categorized based on their BMI at the 11–13‐week assessment, as normal weight (BMI, 18.5–24.9 kg/m^2^), overweight (BMI, 25.0–29.9 kg/m^2^) or obese (BMI, ≥ 30 kg/m^2^).

Details regarding maternal characteristics and the findings of the assessment at 11–13 weeks' gestation were recorded in our electronic database (Viewpoint 5.6; GE Healthcare, Munich, Germany). Data on pregnancy outcomes were obtained from the computerized maternity records or, if needed, from the general medical practitioners. Pre‐eclampsia (PE) was defined according to the American College of Obstetricians and Gynecologists^9^, as chronic or gestational hypertension, with either new‐onset proteinuria or other maternal end‐organ involvement from 20 weeks' gestation. Gestational diabetes mellitus (GDM) was defined as reported previously^10^, ^11^.

### Hemodynamic and vascular measurements

Participants were studied in the supine position after resting for approximately 5 min. Measurement of mean uterine artery pulsatility index (UtA‐PI) was carried out using transabdominal color Doppler ultrasound of the left and right uterine arteries^12^, as a marker for uteroplacental perfusion.

Assessment of the ophthalmic arteries, as a measure of small vessel peripheral circulation, was carried out using a 7.5‐MHz linear transducer (Canon Aplio i900 PLT‐704SBT Linear Probe; Canon Medical Systems Europe BV, Zoetermeer, The Netherlands). Flow velocity waveforms from the left and right ophthalmic arteries were obtained twice in each eye, and the average peak systolic velocity (PSV) ratio of the four measurements was calculated^13^.

Aortic stiffness was assessed directly by measuring carotid‐to‐femoral PWV. Measurements were obtained using the Vicorder device (Skidmore Medical Ltd, Bristol, UK), which measures the time taken for the arterial pulse wave to travel from the carotid to the femoral artery. This device measures simultaneous pressure waveforms by a volume displacement technique, using blood‐pressure cuffs placed around the neck to pick up the carotid pulse wave and the right upper thigh to measure the femoral pulse wave in real time over at least 10 heartbeats. Both cuffs are automatically inflated and the corresponding oscillometric signal is analyzed to accurately measure in real time the pulse time delay and consequent PWV. To calculate transit time, the Vicorder software automatically marks the steepest ascending part of the pulse wave (maximum systolic upstroke) and uses a definite timeframe to detect the nadir of the wave. The shift in time between the marked areas on the carotid and femoral pulse waves, which is the transit time, is detected by cross‐correlation. The distance between the carotid and femoral pressure cuffs was measured using a tape. To account for differences in abdominal circumference associated with pregnancy and to reduce variability and error in distance assessment, all measurements were performed from the suprasternal notch to the right shoulder and from there to the midpoint of the blood pressure cuff on the thigh. PWV was expressed in m/s^14^.

The waveform of brachial artery pulse was also obtained oscillometrically and analyzed using the Vicorder device^15^. By applying a brachial‐to‐aortic generalized transfer function, the aortic waveform was generated. Central systolic and diastolic blood pressure (BP), cardiac output and TPR were derived using the pulse‐wave analysis software. Augmentation pressure was obtained and augmentation index, an indirect measure of aortic stiffness, was expressed as a percentage of central pulse pressure.

### Statistical analysis

Continuous variables were presented as mean ± SD if their distribution was normal, or as median (interquartile range) if their distribution was non‐normal. Normality of the distribution was tested using the Kolmogorov–Smirnov test. Categorical variables were presented as *n* (%).

ANOVA or the Kruskal–Wallis test were used to perform between‐group comparisons of continuous variables, using *post‐hoc* Bonferroni correction to adjust for multiple comparisons when necessary. Chi‐square test or Fisher's exact test were used to perform between‐group comparisons of categorical variables. *P*‐values are presented for the direct two‐way comparisons between BMI groups. *P* < 0.05 was considered statistically significant.

Multivariable regression models were fitted to assess the effect on maternal hemodynamic and vascular measures of BMI (in kg/m^2^) at 11–13 weeks' gestation and gestational weight gain, adjusting for baseline maternal demographics, medical history, pregnancy characteristics and pregnancy outcomes of PE and GDM. Maternal demographics included in the regression model were: maternal age (years), ethnicity (white, black, South Asian, East Asian or mixed) and deprivation (decile of the index of multiple deprivation, across seven domains (https://www.gov.uk/government/statistics/english‐indices‐of‐deprivation‐2019)). Elements of medical history included in the regression model were: prior pregnancy complications (PE, GDM, small‐for‐gestational age or large‐for‐gestational age) and family history of PE or diabetes mellitus (in a first‐degree relative). Pregnancy characteristics included in the regression model were: ovulation induction, *in‐vitro* fertilization (IVF), smoking and nulliparity. Correction for body surface area was not undertaken for cardiac output and TPR measurements, as height and weight were already considered in the multivariable regression analysis.

The statistical software package R (version 2.15.1; The R Foundation for Statistical Computing, Vienna, Austria) was used for data analysis^16^.

## RESULTS

### Participant characteristics

Table 1 presents the baseline characteristics and pregnancy outcomes of the 11 731 women in our study population, according to BMI category; 3403 (29.0%) were overweight and 2136 (18.2%) were obese. Obese women were younger, more often of black ethnicity or deprived, were less likely to have conceived by IVF and were more likely to be a smoker, to have a family history of diabetes mellitus or to have a personal history of pregnancy complication, compared with overweight or normal‐weight women. Obese women gained on average 2.5 kg less weight between 11–13 weeks and 35–37 weeks of gestation, and were more likely to develop PE, gestational hypertension and GDM, compared with overweight or normal‐weight women.

**Table 1:** Maternal and pregnancy characteristics of study population of 11 731 women, according to body mass index (BMI) category at 11–13 weeks' gestation

Characteristic	Normal weight (*n* = 6192)	Overweight (*n* = 3403)	Obese (*n* = 2136)	*P* *	*P* †	*P* ‡
*Maternal characteristics*						
Age (years)	33.6 ± 4.9	33.7 ± 5.1	33.0 ± 5.3	0.977	< 0.001	< 0.001
Ethnicity						
White	4737 (76.5)	2298 (67.5)	1254 (58.7)	< 0.001	< 0.001	< 0.001
Black	548 (8.9)	615 (18.1)	636 (29.8)	< 0.001	< 0.001	< 0.001
South Asian	447 (7.2)	286 (8.4)	132 (6.2)	0.040	0.341	0.002
East Asian	219 (3.5)	48 (1.4)	24 (1.1)	< 0.001	< 0.001	0.758
Mixed	241 (3.9)	156 (4.6)	90 (4.2)	0.565	0.726	0.842
IMD decile	5.71 ± 2.47	5.55 ± 2.60	5.13 ± 2.59	0.010	0.010	0.010
BMI at 11–13 weeks' gestation (kg/m^2^)	22.2 ± 1.7	27.1 ± 1.4	34.6 ± 4.3	< 0.001	< 0.001	< 0.001
Method of conception						
Natural	5653 (91.3)	3140 (92.3)	2013 (94.2)	0.825	0.862	0.884
Ovulation induction	35 (0.6)	19 (0.6)	14 (0.7)	0.872	0.913	0.893
*In‐vitro* fertilization	504 (8.1)	244 (7.2)	109 (5.1)	0.772	< 0.001	< 0.001
Smoker	63 (1.0)	43 (1.3)	42 (2.0)	0.762	0.001	0.043
Family history of PE§	184 (3.0)	108 (3.2)	87 (4.1)	0.972	0.016	0.813
Family history of DM§	630 (10.2)	477 (14.0)	361 (16.9)	< 0.001	< 0.001	< 0.001
Parity						
Nulliparous	3077 (49.7)	1742 (51.2)	1138 (53.3)	0.762	0.004	0.882
Parous, previous PE	108 (1.7)	90 (2.6)	86 (4.0)	< 0.001	< 0.001	< 0.001
Parous, previous SGA	473 (7.6)	256 (7.5)	161 (7.5)	0.843	0.921	0.882
Parous, previous LGA	151 (2.4)	130 (3.8)	131 (6.1)	< 0.001	< 0.001	< 0.001
Parous, previous GDM	142 (2.3)	133 (3.9)	151 (7.1)	< 0.001	< 0.001	< 0.001
*Pregnancy characteristics*						
Maternal weight gain from 11–13 to 35–37 weeks (kg)	11.1 ± 4.3	10.2 ± 4.7	7.7 ± 4.3	< 0.001	< 0.001	< 0.001
PE	141 (2.3)	91 (2.7)	113 (5.3)	< 0.001	< 0.001	< 0.001
Gestational hypertension	189 (3.1)	149 (4.4)	141 (6.6)	< 0.001	< 0.001	< 0.001
GDM	439 (7.1)	417 (12.3)	476 (22.3)	< 0.001	< 0.001	< 0.001
Birth‐weight percentile	46 ± 28	51 ± 29	53 ± 29	< 0.001	< 0.001	0.002

Data are given as mean ± SD or *n* (%).

BMI was categorized as normal (18.5–24.9 kg/m^2^), overweight (25.0–29.9 kg/m^2^) or obese (≥ 30.0 kg/m^2^).

* Comparison between overweight and normal weight.

† Comparison between obese and normal weight.

‡ Comparison between obese and overweight.

§ First‐degree relative.

DM, diabetes mellitus; GDM, gestational diabetes mellitus; IMD, index of multiple deprivation (decile 1 is most deprived, decile 10 is least deprived); LGA, large‐for‐gestational age; PE, pre‐eclampsia, SGA, small‐for‐gestational age.

### Maternal hemodynamic and vascular indices and UtA‐PI at 11–13 weeks

Table 2 presents the maternal cardiovascular parameters and UtA‐PI for each BMI category. Overweight and obese women had higher central systolic and diastolic BP, indices of aortic stiffness (augmentation index and carotid‐to‐femoral PWV), cardiac output and UtA‐PI, compared with normal‐weight women. There was no significant difference between groups in TPR or ophthalmic artery PSV ratio. A similar pattern was seen in the comparison between obese and overweight women, with increased parameters in the obese group.

**Table 2:** Maternal cardiovascular parameters and uterine artery pulsatility index (UtA‐PI) at 35–37 weeks' gestation, according to body mass index (BMI) category at 11–13 weeks' gestation

Parameter	Normal weight (*n* = 6192)	Overweight (*n* = 3403)	Obese (*n* = 2136)	*P* *	*P* †	*P* ‡
SBP (mmHg)	112 ± 10	115 ± 11	120 ± 11	< 0.001	< 0.001	< 0.001
DBP (mmHg)	63 ± 7	65 ± 7	67 ± 8	< 0.001	< 0.001	< 0.001
AIx@75 (%)	26 ± 14	29 ± 13	30 ± 13	< 0.001	< 0.001	< 0.001
CO (L/min)	6.93 ± 1.46	7.14 ± 1.56	7.61 ± 1.84	< 0.001	< 0.001	< 0.001
Carotid‐to‐femoral PWV (m/s)	8.38 ± 1.27	8.63 ± 1.34	8.90 ± 1.55	< 0.001	< 0.001	< 0.001
TPR (mmHg × min/L)	0.78 ± 0.17	0.77 ± 0.17	0.77 ± 0.19	0.915	0.944	0.966
OA‐PSV ratio	0.62 ± 0.10	0.61 ± 0.11	0.61 ± 0.11	0.858	0.785	0.985
UtA‐PI	47 ± 30	49 ± 30	52 ± 30	0.001	0.001	0.006

Data are given as mean ± SD.

BMI was categorized as normal (18.5–24.9 kg/m^2^), overweight (25.0–29.9 kg/m^2^) or obese (≥ 30.0 kg/m^2^).

* Comparison between overweight and normal weight.

† Comparison between obese and normal weight.

‡ Comparison between obese and overweight.

AIx@75, augmentation index adjusted for heart rate of 75 bpm; CO, cardiac output; DBP, central diastolic blood pressure; OA‐PSV, ophthalmic artery peak systolic velocity; PWV, pulse‐wave velocity; SBP, central systolic blood pressure; TPR, total peripheral resistance.

### Association of BMI at 11–13 weeks and weight gain with hemodynamic and vascular indices

Table 3 reports the determinants of maternal hemodynamic and vascular indices at 35–37 weeks' gestation. Independent of maternal demographics, medical history, pregnancy characteristics and pregnancy outcomes, increasing BMI at 11–13 weeks' gestation (considered as a continuous variable in kg/m^2^) was associated with increased cardiac output, aortic stiffness (assessed by augmentation index and carotid‐to‐femoral PWV), ophthalmic artery PSV ratio and UtA‐PI at 35–37 weeks, but there was no association with TPR. Additionally, increasing gestational weight gain between 11–13 weeks and 35–37 weeks was independently associated with increased cardiac output, aortic stiffness (assessed by augmentation index and carotid‐to‐femoral PWV), ophthalmic artery PSV ratio and UtA‐PI measured at 35–37 weeks, but was associated with decreased TPR. These associations were independent of the multiple other associations observed between the hemodynamic and vascular indices assessed and maternal demographics (maternal age, ethnicity), prior pregnancy complications, family history of PE or diabetes mellitus, pregnancy characteristics (IVF, smoking) and pregnancy outcomes of PE and GDM.

**Table 3:** Association of maternal hemodynamic and vascular indices and uterine artery pulsatility index at 35–37 weeks' gestation with body mass index (BMI) at 11–13 weeks, gestational weight gain and maternal/pregnancy characteristics

Variable	Augmentation index* (%)		Cardiac output (L/min)		Carotid‐to‐femoral PWV (m/s)		Total peripheral resistance (mmHg × min/L)		Ophthalmic artery PSV ratio		Uterine artery pulsatility index	
	Beta (95% CI)	*P*	Beta (95% CI)	*P*	Beta (95% CI)	*P*	Beta (95% CI)	*P*	Beta (95% CI)	*P*	Beta (95% CI)	*P*
BMI at 11–13 weeks†	0.41 (0.36–0.46)	< 0.001	0.05 (0.04–0.05)	< 0.001	0.04 (0.04–0.05)	< 0.001	−0.0002 (−0.0008 to 0.0004)	0.55	0.0005 (0.0001–0.0008)	0.02	0.35 (0.24–0.46)	< 0.001
Gestational weight gain‡	0.22 (0.16–0.27)	< 0.001	0.03 (0.02–0.03)	< 0.001	0.01 (0.01–0.02)	< 0.001	−0.0013 (−0.0020 to −0.0007)	< 0.001	0.0004 (0.0002–0.0006)	0.03	0.23 (0.11–0.35)	< 0.001
*Demographics and medical history*												
Maternal age§	0.00 (−0.05 to 0.05)	0.92	−0.08 (−0.09 to −0.08)	< 0.001	0.05 (0.05–0.06)	< 0.001	0.01 (0.01–0.01)	< 0.001	0.0042 (0.0038–0.0046)	< 0.001	0.48 (0.37–0.60)	<0.001
Ethnicity¶												
Black	−3.20 (−3.94 to −2.46)	< 0.001	0.19 (0.11–0.27)	< 0.001	0.04 (−0.03 to 0.11)	0.36	−0.02 (−0.03 to −0.01)	< 0.001	−0.04 (−0.04 to −0.03)	< 0.001	4.27 (2.67–5.88)	< 0.001
South Asian	4.33 (3.36–5.30)	< 0.001	0.01 (−0.09 to 0.12)	0.79	0.15 (0.05–0.24)	0.002	−0.007 (−0.019 to 0.004)	0.22	−0.0010 (−0.0083 to 0.0062)	0.77	1.42 (−0.69 to 3.53)	0.18
East Asian	3.19 (1.64–4.74)	< 0.001	−0.15 (−0.32 to 0.02)	0.09	0.28 (0.13–0.43)	< 0.001	0.01 (−0.01 to 0.03)	0.44	−0.03 (−0.04 to −0.02)	< 0.001	−1.96 (−5.35 to 1.43)	0.25
Mixed	0.23 (−1.01 to 1.48)	0.71	0.03 (−0.11 to 0.16)	0.71	0.01 (−0.11 to 0.13)	0.84	−0.01 (−0.02 to 0.01)	0.40	−0.01 (−0.02 to −0.01)	0.003	2.28 (−0.43 to 4.99)	0.10
Deprivation	−0.07 (−0.17 to 0.03)	0.13	−0.0001 (−0.0111 to 0.0109)	0.98	0.0050 (−0.0047 to 0.0147)	0.30	0.0000 (−0.0013 to 0.0012)	0.96	−0.0004 (−0.0011 to 0.0004)	0.31	−0.02 (−0.24 to 0.20)	0.83
Prior PE	−1.05 (−2.68 to 0.56)	0.20	0.29 (0.11–0.46)	0.002	0.32 (0.16–0.47)	0.003	−0.01 (−0.03 to 0.01)	0.36	0.02 (0.004–0.028)	0.007	3.11 (−0.41 to 6.63)	0.08
Prior FGR	0.23 (−0.71 to 1.16)	0.63	−0.02 (−0.12 to 0.09)	0.74	0.04 (−0.05 to 0.13)	0.39	0.0032 (−0.0082 to 0.0146)	0.58	0.02 (0.01–0.02)	< 0.001	2.86 (0.82–4.89)	0.006
Prior GDM	−0.20 (−1.57 to 1.16)	0.77	0.25 (0.10–0.40)	0.001	0.03 (−0.11 to 0.16)	0.74	−0.03 (−0.04 to −0.01)	0.003	−0.0087 (−0.0189 to 0.0015)	0.10	2.40 (−0.58 to 5.37)	0.12
Prior LGA	−1.08 (−2.43 to 0.27)	0.11	0.31 (0.16–0.45)	< 0.001	0.08 (−0.05 to 0.22)	0.22	−0.03 (−0.05 to −0.02)	< 0.001	−0.03 (−0.04 to −0.02)	< 0.001	1.99 (−0.96 to 4.94)	0.18
Family history of PE**	0.61 (−0.78 to 2.00)	0.38	0.01 (−0.14 to 0.16)	0.89	0.06 (−0.08 to 0.19)	0.39	0.0175 (0.0006–0.0345)	0.04	0.0041 (−0.0063 to 0.0145)	0.43	−0.79 (−3.83 to 2.24)	0.60
Family history of DM**	0.16 (−0.60 to 0.92)	0.68	0.06 (−0.02 to 0.14)	0.16	0.03 (−0.04 to 0.10)	0.40	0.0043 (−0.0050 to 0.0137)	0.36	−0.0027 (−0.0084 to 0.0030)	0.34	1.95 (0.28–3.61)	0.02
*Pregnancy characteristics and outcomes*												
Ovulation induction	−2.19 (−5.32 to 0.95)	0.17	−0.19 (−0.53 to 0.15)	0.27	−0.16 (−0.46 to 0.14)	0.30	0.02 (−0.02 to 0.05)	0.42	−0.0021 (−0.0255 to 0.0214)	0.86	−1.50 (−8.35 to 5.35)	0.66
IVF	−0.53 (−1.50 to 0.44)	0.28	−0.03 (−0.14 to 0.07)	0.55	−0.03 (−0.13 to 0.06)	0.47	0.02 (0.01–0.03)	< 0.001	0.0100 (0.0027–0.0173)	0.007	−6.32 (−8.44 to −4.20)	< 0.001
Smoker	1.89 (−0.29 to 4.07)	0.09	−0.05 (−0.28 to 0.19)	0.71	−0.11 (−0.32 to 0.10)	0.29	0.0217 (−0.0049 to 0.0482)	0.11	0.0201 (0.0038–0.0364)	0.02	3.28 (−1.48 to 8.04)	0.17
Nulliparous	−0.12 (−0.60 to 0.37)	0.64	−0.03 (−0.08 to 0.03)	0.33	−0.03 (−0.08 to 0.02)	0.17	0.0003 (−0.0057 to 0.0062)	0.93	−0.0020 (−0.0056 to 0.0017)	0.29	−0.38 (−1.45 to 0.68)	0.48
PE	0.81 (−0.65 to 2.27)	0.27	0.29 (0.13–0.45)	< 0.001	0.94 (0.80–1.08)	< 0.001	0.04 (0.04–0.06)	< 0.001	0.08 (0.07–0.09)	<0.001	7.20 (4.02–10.37)	< 0.001
GDM	0.77 (−0.05 to 1.60)	0.07	0.10 (0.02–0.19)	0.02	0.11 (0.03–0.19)	0.006	0.005 (−0.005 to 0.02)	0.29	0.01 (0.006–0.02)	<0.001	1.10 (−0.69 to 2.90)	0.23

* At 75 bpm.

† Per 1‐kg/m^2^ increase.

‡ Between 11–13 weeks and 35–37 weeks, per 1‐kg increase.

§ Per 1‐year increase.

¶ White ethnicity was used as reference.

** First‐degree relative.

DM, diabetes mellitus; FGR, fetal growth restriction; GDM, gestational diabetes mellitus; IVF, *in‐vitro* fertilization; LGA, large‐for‐gestational age; PE, pre‐eclampsia; PSV, peak systolic velocity; PWV, pulse‐wave velocity.

## DISCUSSION

### Main findings

In this large screening study of almost 12 000 women with a singleton pregnancy undergoing routine assessment at 35–37 weeks' gestation, overweight and obese women had altered hemodynamic and vascular indices compared with normal‐weight women. Specifically, overweight and obese women had increased central systolic and diastolic BP, cardiac output, aortic stiffness (as measured by augmentation index and carotid‐to‐femoral PWV) and UtA‐PI. On multivariable analysis considering BMI as a continuous variable, increasing BMI was associated with increased ophthalmic artery PSV ratio. There was no demonstrable difference in TPR between BMI groups in any analysis. Importantly, increased gestational weight gain between 11–13 weeks and 35–37 weeks was independently associated with altered maternal hemodynamics and vascular indices in the third trimester, as were multiple maternal and pregnancy characteristics, including pregnancy outcome of PE or GDM.

Our findings suggest that increased BMI is associated with altered hemodynamic and vascular indices independent of pregnancy outcome, including PE. These changes may represent pre‐existing alterations in maternal physiology and/or a maladaptation to pregnancy. Regardless, the data suggest that optimization of both prepregnancy BMI and gestational weight gain could potentially improve cardiovascular health in pregnant women, and therefore may improve pregnancy outcomes.

### Interpretation of results and comparison with previous studies

Obesity has long been considered a risk factor for adverse cardiovascular outcomes. Large longitudinal studies have demonstrated that increased maternal prepregnancy BMI increases the risk of subsequent development of diabetes, hypertension and coronary heart disease in the mother^17^, ^18^, ^19^, ^20^, ^21^, ^22^. It is also well‐reported that women with increased prepregnancy BMI are at higher risk of developing pregnancy complications^23^, ^24^. Consistent with these findings, we have shown that overweight and obese pregnant women were more likely to develop gestational hypertension, PE and GDM compared with normal‐weight women. However, it is unclear what mediates the relationship between high BMI and adverse pregnancy outcomes; possibilities include the underlying maternal risk‐factor profile, metabolic and inflammatory profiles, and hemodynamic status at baseline or in response to pregnancy.

In the current study, overweight and obese (*vs* normal‐weight) women had increased association with vascular disease parameters. Aortic stiffness was increased, as assessed by carotid‐to‐femoral PWV and augmentation index. In a general population, aortic stiffness is strongly associated with a higher risk of cardiovascular disease, which increases with age, and is often apparent before the development of clinical arterial hypertension^25^. Development of arterial stiffness is multifactorial; it is driven by the production of endocrine factors and cytokines, as well as by interactions between different vascular cellular components^26^, ^27^, ^28^. Visceral obesity is more closely linked with arterial stiffness than with BMI, as arterial stiffness has been found to be associated with insulin resistance, increased inflammation and increased production of leptin, which induces oxidative stress in endothelial cells and triggers the transcription of oxidant‐sensitive genes that participate in atherogenesis^29^, ^30^.

Apart from vascular disease parameters, increased BMI was also associated with hemodynamic alterations at 35–37 weeks' gestation in our study. Previous studies of maternal hemodynamics in obese women are limited in participant numbers, to a combined total of 354 obese women and 1300 normal‐weight women, and provide conflicting results. A cross‐sectional study of pregnant woman at term reported that 40 women with BMI ≥ 35 kg/m^2^ (*vs* 40 with normal BMI) had higher systolic BP, cardiac output, left ventricular mass index and relative wall thickness, as well as reduced diastolic function^6^. In another study at 31 weeks' gestation, 23 women with BMI ≥ 40 kg/m^2^ (*vs* 327 with normal BMI) had lower stroke volume index and cardiac index, and higher TPR index, after correction for body surface area^31^. Similarly, in a longitudinal study, 232 women with BMI ≥ 35 kg/m^2^ (*vs* 919 with normal BMI) had lower cardiac output and disappearance of the low TPR, which is typical of pregnancy, in the third trimester^7^. In another longitudinal study in the third trimester of pregnancy, 59 women with BMI ≥ 35 kg/m^2^ (*vs* 14 with normal BMI) had higher cardiac output and lower TPR, but there was no significant difference between BMI groups with regard to gestational weight gain^32^. The discrepancy in results between reported studies is likely to reflect: differences in demographics between study populations; lack of adjustment for maternal characteristics; correction for only body surface area when assessing maternal hemodynamics, without accounting for other demographic parameters; differences in gestational weight gain; and/or differences in adverse pregnancy outcomes associated with hemodynamic and vascular changes, including GDM^10^ and PE, for which the changes may precede clinical disease^15^.

### Clinical implications

The results of our study confirm that both BMI at 11–13 weeks and gestational weight gain from the first to the third trimester independently affect hemodynamic and vascular indices at 35–37 weeks' gestation, and these relationships are not accounted for by GDM or subclinical changes related to PE. These findings argue that optimization of maternal weight, both before and during pregnancy, may be important in promoting arterial health, in addition to promoting a generally healthy lifestyle and cardiovascular risk reduction.

### Strengths and limitations

This study consisted of a large population‐based sample for which we had obtained detailed information on participant characteristics, which allowed us to account for a variety of possible confounders of the relationship between BMI and both hemodynamic and vascular indices in our analysis. Our study population was diverse, with regard to ethnicity and socioeconomic background, improving the generalizability of our results. We had recorded accurate information on both maternal BMI at 11–13 weeks' gestation and gestational weight gain between 11–13 weeks and 35–37 weeks, which allowed us to examine their independent effects on hemodynamic and vascular measures. We performed detailed phenotyping to characterize both maternal hemodynamics and central and peripheral vasculature. We used non‐invasive, operator‐independent techniques to improve the reproducibility of our results and improve ease of assessment.

Limitations of the study include the use of BMI at 11–13 weeks as a proxy for prepregnancy BMI. Whilst BMI is a simple tool which is used routinely in clinical practice, we recognize its limitations, in that it does not distinguish between different types of body fat and includes the weight of all body components, including muscle, bone and water weight. We did not obtain any additional biochemical or imaging measurements to characterize visceral adiposity. Vascular assessment was performed only in the third trimester, and not serially throughout pregnancy. We did not obtain any prepregnancy information on vascular status, therefore, we were unable to determine whether our findings reflect obesity‐related prepregnancy vascular dysfunction, or hemodynamic and vascular maladaptation to pregnancy. In addition, cardiac output was estimated using the pulse‐wave analysis software associated with the Vicorder device, rather than through echocardiography.

### Conclusions

Our study demonstrates that women with a high BMI in early pregnancy (compared with normal‐weight women) and/or those with higher gestational weight gain had worse maternal hemodynamic and vascular indices at 35–37 weeks' gestation, and these findings are not accounted for by outcomes of PE or GDM. Our findings support the optimization of prepregnancy weight and gestational weight gain. Future research should address whether the hemodynamic changes observed in this study are part of the causal pathway between obesity and adverse pregnancy outcomes and may help to identify obese women at increased risk of pregnancy complications.

## Data Availability

Research data are not shared.
